# 4-Acetamido-3-chloro­phenyl acetate

**DOI:** 10.1107/S2414314626000763

**Published:** 2026-01-29

**Authors:** Sindhu V. Bai, Guoqiang Li, Patrick F. Mensah, Frank R. Fronczek, Rao M. Uppu

**Affiliations:** a Department of Environmental Toxicology Southern University and A&M College,Baton Rouge Louisiana 70813 USA; b Department of Mechanical & Industrial Engineering Louisiana State University,Baton Rouge Louisiana 70803 USA; c Department of Mechanical Engineering Southern University and A&M College, Baton Rouge Louisiana 70813 USA; d Department of Chemistry Louisiana State University,Baton Rouge Louisiana 70803 USA; University of Aberdeen, United Kingdom

**Keywords:** crystal structure, chlorinated acetamino­phen, hypochlorite–hypo­chlorous acid oxidation

## Abstract

In the title compound, C_10_H_10_ClNO_3_, the dihedral angles between the chloro­benzene ring and the acetamide and acetate planes are 40.70 (8) and 88.07 (8)°, respectively; the acetamide and acetate planes make a dihedral angle of 51.39 (9)°. In the extended structure, the mol­ecules are linked by N—H⋯O hydrogen bonds involving the acetamide group, forming *C*(4) chains propagating along the [010] direction.

## Abstract 2

In the title compound, the dihedral angles between the chloro­benzene ring and the acetamide and acetate planes are 40.70 (8) and 88.07 (8)°, respectively; the acetamide and acetate planes make a dihedral angle of 51.39 (9)°. In the extended structure, the mol­ecules are linked by N—H⋯O hydrogen bonds involving the acetamide group, forming *C*(4) chains propagating along the [010] direction.

## Graphical abstract

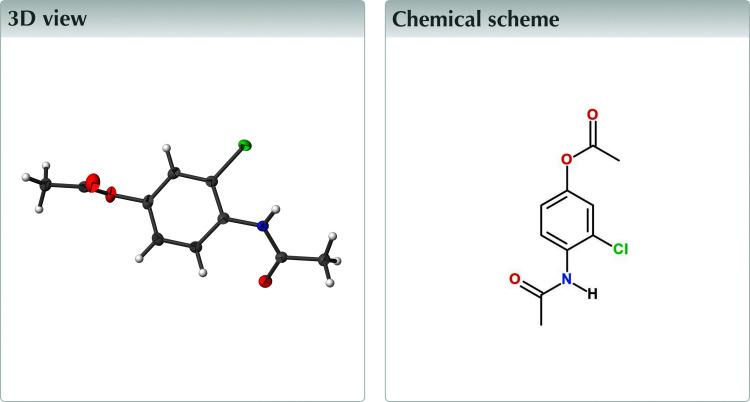

## Structure description

With approximately 25 billion doses sold annually in the United States, acetamino­phen, C_8_H_9_NO_2_ (also known as paracetamol; brand names in different countries include Tylenol, Panadol and many others) is among the most widely used analgesic and anti­pyretic agents (Uppu & Fronczek, 2025 ▸; Yoon *et al.*, 2016 ▸). Its metabolism is dominated by sulfation and glucuronidation, with a smaller contribution from CYP2E1-mediated oxidation (Maza­leuskaya *et al.*, 2015 ▸). In addition, non-enzymatic transformations mediated by cellular oxidants, including the per­oxy­nitrite–CO_2_ system and the myeloperoxidase–H_2_O_2_–Cl^−^ pathway that generates HOCl/ClO^−^ (p*K*_a_ ≃ 7.53), warrant consideration (Bedner & MacCrehan, 2006 ▸; Hines *et al.*, 2025 ▸; Hines *et al.*, 2026 ▸; Uppu & Martin, 2005 ▸). While CYP2E1 and related oxidants are implicated in overdose-level formation of *N*-acetyl-1,4-benzo­quinone imine (NBQI), the present work focuses on low-level *in vivo* NBQI formation and the identification of chemically tractable transformation products (Manyike *et al.*, 2000 ▸).

Given the high intra­cellular abundance of chloride ions (*ca*. 150 m*M*), *N*-(4-hy­droxy-2-chloro­phen­yl)acetamide (the 2-chloro isomer; Matsuno *et al.*, 1989 ▸) has been proposed as a chemically plausible product of NBQI–Cl^−^ chemistry. Recognizing that *O*-acetyl­ation can facilitate cellular uptake, we synthesized the title compound, C_10_H_10_ClNO_3_ (**I**), the *O*-acetyl­ated derivative of *N*-(4-hy­droxy-2-chloro­phen­yl)acetamide, and determined its crystal structure (Bai *et al.*, 2025 ▸).

**Figure 1:**
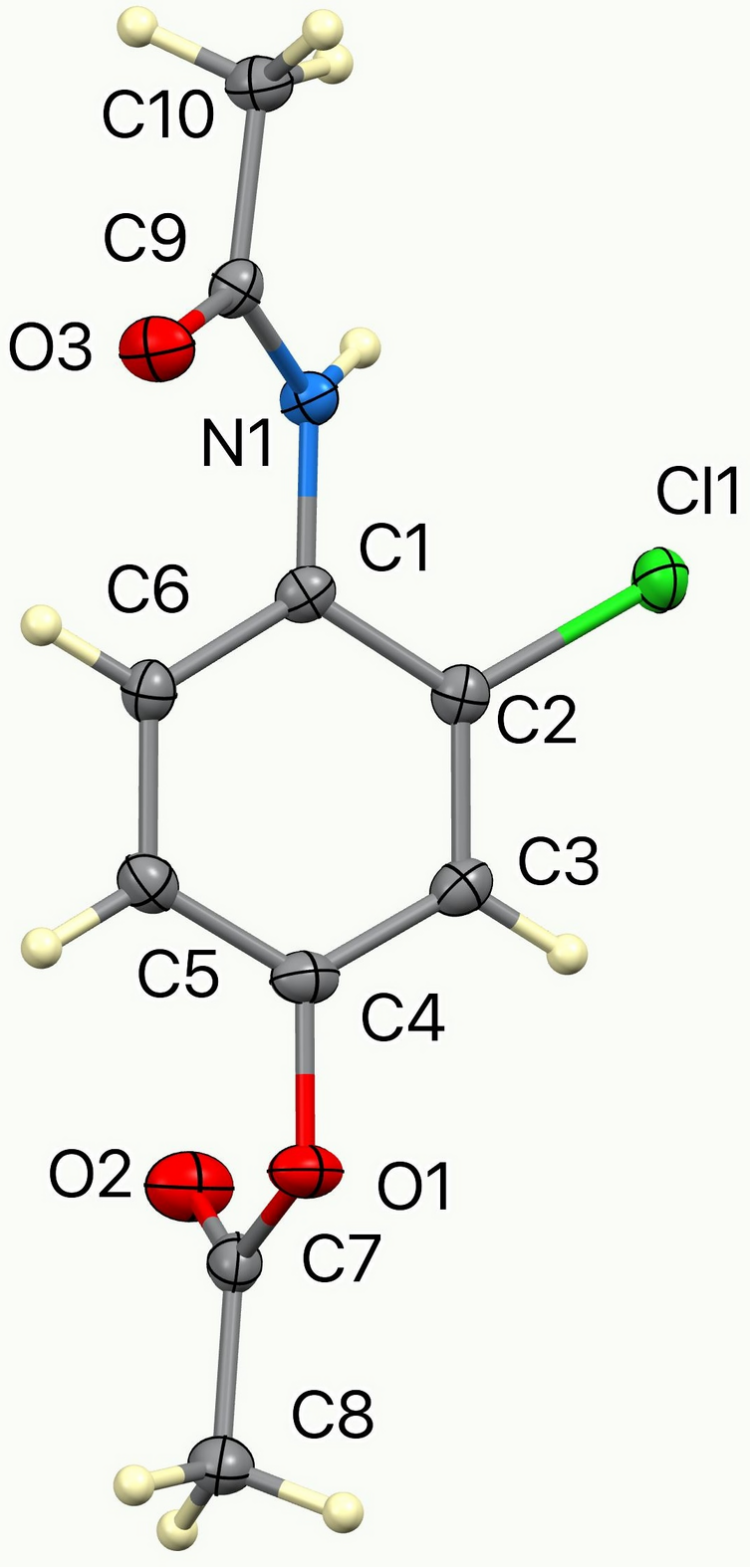
The mol­ecular structure of (**I**) with displacement ellipsoids drawn at the 50% probability level.

**Figure 2:**
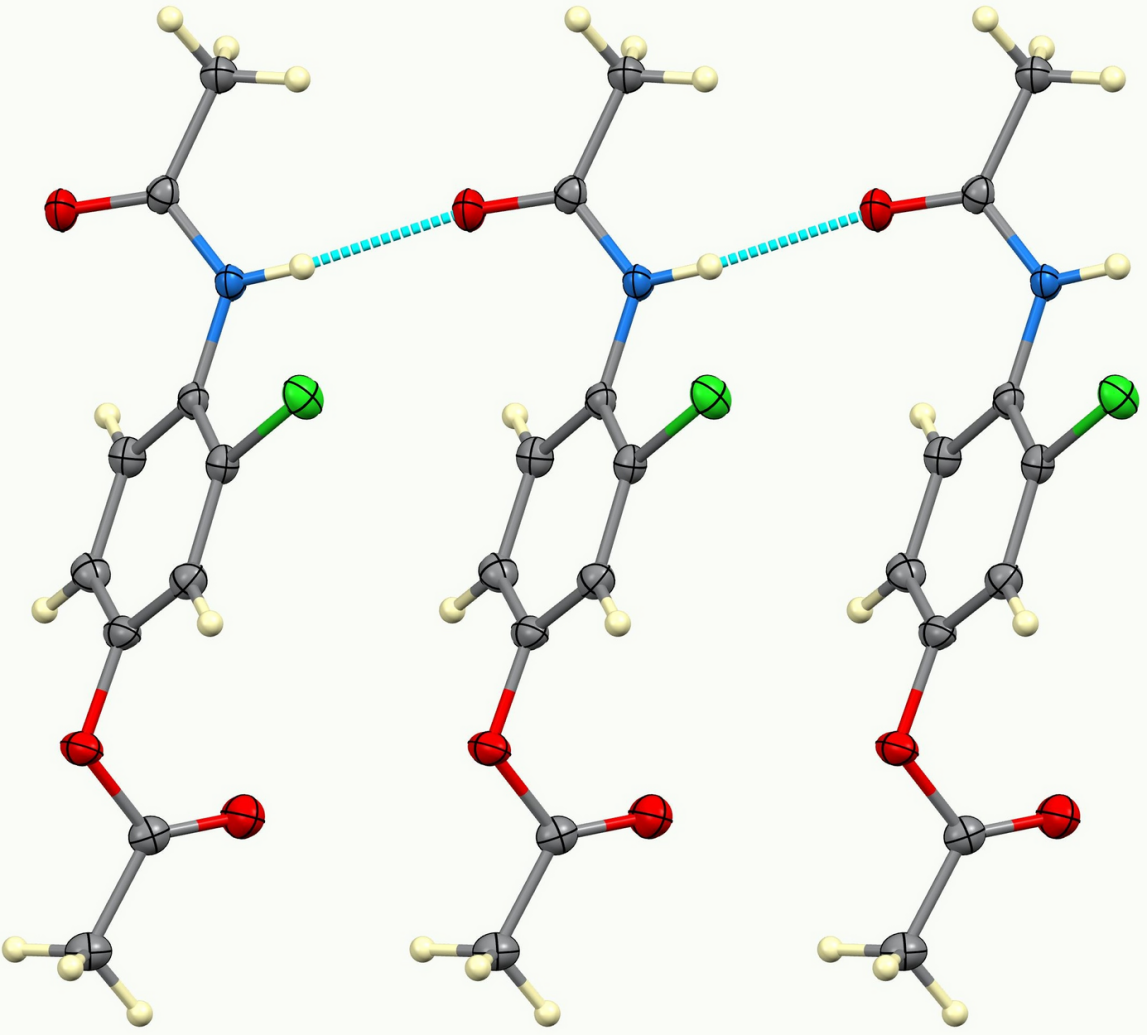
Fragment of a [010] hydrogen-bonded chain in (**I**).

**Figure 3:**
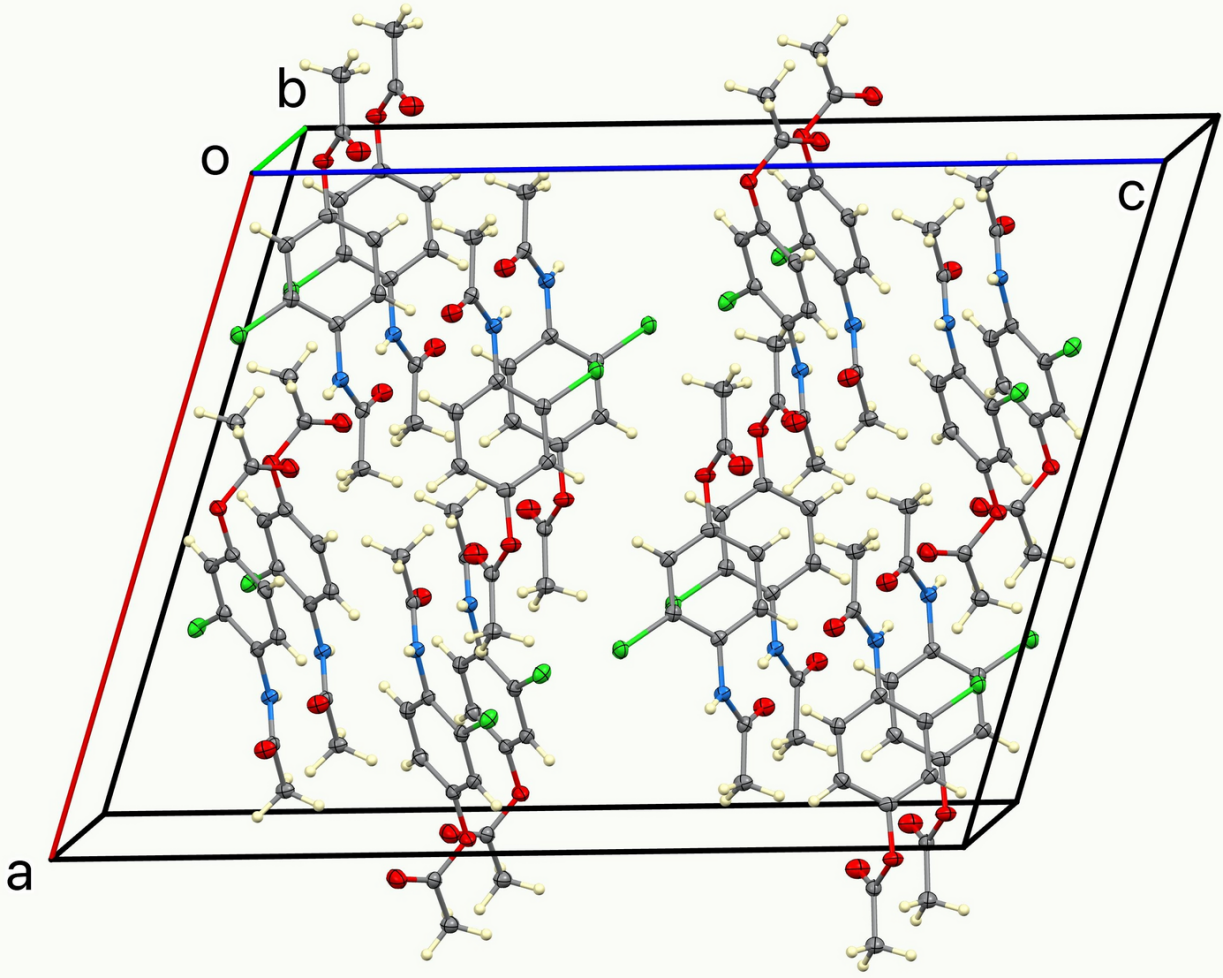
The unit cell of (**I**) viewed approximately down [010].

Compound (**I**) crystallizes as a neutral mol­ecular species in space group *I*2/*a* with one mol­ecule in the asymmetric unit (Fig. 1 ▸). The dihedral angles between the central C1–C6 aromatic ring and the pendant acetamide (C9/C10/N1/O3) and acetate (C7/C8/O1/O2) mean planes are 40.70 (8) and 88.07 (8)°, respectively: the two substituents are displaced on opposite sides of the central ring. The masking of the phenolic O—H group as an ester in (**I**) suppresses phenol-based hydrogen bonding and shifts the supra­molecular assembly to an amide-centered hydrogen-bonding network in which N1—H1*N*⋯O3 hydrogen bonds link the mol­ecules into *C*(4) chains propagating in the [010] direction with adjacent mol­ecules in the chain related by simple translation (Table 1 ▸, Fig. 2 ▸). Weak C—H⋯Cl and C—H⋯O inter­actions consolidate the structure (Fig. 3 ▸). This solid-state behavior is consistent with an ester that is poised for enzymatic *O*-de­acetyl­ation to *N*-(4-hy­droxy-2-chloro­phen­yl)acetamide (Soloviev *et al.*, 2022 ▸). The mol­ecular geometry and packing parameters reported here provide a crystallographic reference for comparison with related acetamino­phen derivatives and their functionalized analogues.

## Synthesis and crystallization

The title compound was synthesized by acetyl­ation of 4-amino-3-chloro­phenol with acetic anhydride following Naik *et al.* (2004 ▸) with minor modifications: 4-amino-3-chloro­phenol hydro­chloride (1.8 g, 10 mmol) was dissolved in ∼50–75 ml water and adjusted to pH 1.5–1.7 with 1.0 *N* HCl. The cooled solution (ice bath) was treated with acetic anhydride (1.21 ml, 12 mmol), then sodium bicarbonate (2.16–3.02 g, 25–35 mmol) was added with continuous stirring, maintaining the reaction pH between 5.5 and 6.5. The off-white precipitate was collected by filtration. Crystals were grown from a hot, near-saturated ethano­lic aqueous solution by slow cooling and evaporation to form colorless needles of (**I**).

## Refinement

Crystal data, data collection and structure refinement details are summarized in Table 2 ▸.

## Supplementary Material

Crystal structure: contains datablock(s) I. DOI: 10.1107/S2414314626000763/hb4554sup1.cif

Structure factors: contains datablock(s) I. DOI: 10.1107/S2414314626000763/hb4554Isup2.hkl

Supporting information file. DOI: 10.1107/S2414314626000763/hb4554Isup3.cml

CCDC reference: 2492307

Additional supporting information:  crystallographic information; 3D view; checkCIF report

## Figures and Tables

**Table 1:** Hydrogen-bond geometry (Å, °)

*D*—H⋯*A*	*D*—H	H⋯*A*	*D*⋯*A*	*D*—H⋯*A*
N1—H1*N*⋯O3^i^	0.87 (2)	2.01 (2)	2.8335 (17)	157.5 (17)
C8—H8*A*⋯Cl1^ii^	0.98	2.90	3.8279 (16)	159
C8—H8*C*⋯Cl1^iii^	0.98	2.97	3.6541 (16)	128
C10—H10*A*⋯O3^i^	0.98	2.46	3.2796 (18)	141

Symmetry codes: (i) 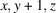; (ii) 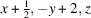; (iii) 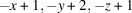.

**Table 2:** Experimental details

Crystal data	
Chemical formula	C_10_H_10_ClNO_3_
*M*_r_	227.64
Crystal system, space group	Monoclinic, *I*2/*a*
Temperature (K)	100
*a*, *b*, *c* (Å)	19.2482 (7), 4.7223 (2), 24.3718 (11)
β (°)	111.222 (2)
*V* (Å^3^)	2065.06 (15)
*Z*	8
Radiation type	Cu *K*α
μ (mm^−1^)	3.19
Crystal size (mm)	0.40 × 0.03 × 0.02

Data collection	
Diffractometer	Bruker D8 Venture DUO with Photon III C14
Absorption correction	Multi-scan (*SADABS*; Krause *et al.*, 2015 ▸)
*T*_min_, *T*_max_	0.795, 0.939
No. of measured, independent and observed [*I* > 2σ(*I*)] reflections	20794, 2190, 1953
*R*_int_	0.049
(sin θ/λ)_max_ (Å^−1^)	0.638

Refinement	
*R*[*F*^2^ > 2σ(*F*^2^)], *wR*(*F*^2^), *S*	0.031, 0.079, 1.06
No. of reflections	2190
No. of parameters	141
H-atom treatment	H atoms treated by a mixture of independent and constrained refinement
Δρ_max_, Δρ_min_ (e Å^−3^)	0.28, −0.31

Computer programs: *APEX5* and *SAINT* (Bruker, 2016 ▸), *SHELXT2018/2* (Sheldrick, 2015*a* ▸), *SHELXL2019/1* (Sheldrick, 2015*b* ▸) and *Mercury* (Macrae *et al.*, 2020 ▸).

## full crystallographic data

### 4-Acetamido-3-chlorophenyl acetate Crystal data

**Table:**

C_10_H_10_ClNO_3_	*F*(000) = 944
*M**_r_* = 227.64	*D*_x_ = 1.464 Mg m^−^^3^
Monoclinic, *I*2/*a*	Cu *K*α radiation, λ = 1.54184 Å
*a* = 19.2482 (7) Å	Cell parameters from 7338 reflections
*b* = 4.7223 (2) Å	θ = 5.1–78.8°
*c* = 24.3718 (11) Å	µ = 3.19 mm^−^^1^
β = 111.222 (2)°	*T* = 100 K
*V* = 2065.06 (15) Å^3^	Needle, colourless
*Z* = 8	0.40 × 0.03 × 0.02 mm

### 4-Acetamido-3-chlorophenyl acetate Data collection

**Table:**

Bruker D8 Venture DUO with Photon III C14 diffractometer	1953 reflections with *I* > 2σ(*I*)
Radiation source: IµS 3.0 microfocus	*R*_int_ = 0.049
φ and ω scans	θ_max_ = 79.5°, θ_min_ = 3.9°
Absorption correction: multi-scan (SADABS; Krause *et al.*, 2015)	*h* = −23→24
*T*_min_ = 0.795, *T*_max_ = 0.939	*k* = −5→5
20794 measured reflections	*l* = −30→30
2190 independent reflections	

### 4-Acetamido-3-chlorophenyl acetate Refinement

**Table:**

Refinement on *F*^2^	0 restraints
Least-squares matrix: full	Hydrogen site location: mixed
*R*[*F*^2^ > 2σ(*F*^2^)] = 0.031	H atoms treated by a mixture of independent and constrained refinement
*wR*(*F*^2^) = 0.079	*w* = 1/[σ^2^(*F*_o_^2^) + (0.0352*P*)^2^ + 2.3982*P*] where *P* = (*F*_o_^2^ + 2*F*_c_^2^)/3
*S* = 1.06	(Δ/σ)_max_ = 0.001
2190 reflections	Δρ_max_ = 0.28 e Å^−^^3^
141 parameters	Δρ_min_ = −0.31 e Å^−^^3^

### 4-Acetamido-3-chlorophenyl acetate Special details

**Table:**

Geometry. All esds (except the esd in the dihedral angle between two l.s. planes) are estimated using the full covariance matrix. The cell esds are taken into account individually in the estimation of esds in distances, angles and torsion angles; correlations between esds in cell parameters are only used when they are defined by crystal symmetry. An approximate (isotropic) treatment of cell esds is used for estimating esds involving l.s. planes.
Refinement. The N-bound H atom was located in a difference map and its positon was freely refined. The C-bound H atoms were geometrically placed (C—H = 0.95–0.98 Å) and refined as riding atoms. The constraint *U*_iso_(H) = 1.2*U*_eq_(C) or 1.5*U*_eq_(methyl C or N) was applied in all cases.

### 4-Acetamido-3-chlorophenyl acetate Fractional atomic coordinates and isotropic or equivalent isotropic displacement parameters (Å^2^)

**Table:**

	*x*	*y*	*z*	*U*_iso_*/*U*_eq_	
Cl1	0.29945 (2)	1.05930 (8)	0.43887 (2)	0.02274 (12)	
O1	0.51347 (6)	0.4581 (2)	0.42785 (5)	0.0234 (2)	
O2	0.54882 (6)	0.7934 (3)	0.37753 (5)	0.0311 (3)	
O3	0.16631 (6)	0.3467 (2)	0.29322 (5)	0.0240 (2)	
N1	0.21473 (7)	0.7825 (3)	0.32425 (5)	0.0166 (3)	
H1N	0.2050 (11)	0.961 (4)	0.3252 (8)	0.025*	
C1	0.29024 (8)	0.6998 (3)	0.34951 (6)	0.0164 (3)	
C2	0.33611 (8)	0.8169 (3)	0.40297 (6)	0.0175 (3)	
C3	0.41082 (8)	0.7434 (3)	0.42888 (6)	0.0193 (3)	
H3	0.441580	0.827008	0.464947	0.023*	
C4	0.43917 (8)	0.5462 (3)	0.40099 (6)	0.0198 (3)	
C5	0.39561 (8)	0.4239 (3)	0.34806 (7)	0.0206 (3)	
H5	0.416175	0.287458	0.329626	0.025*	
C6	0.32145 (8)	0.5039 (3)	0.32240 (6)	0.0189 (3)	
H6	0.291440	0.423692	0.285717	0.023*	
C7	0.56449 (8)	0.5943 (3)	0.41004 (6)	0.0196 (3)	
C8	0.63895 (8)	0.4550 (4)	0.43598 (7)	0.0236 (3)	
H8A	0.675565	0.561234	0.424852	0.035*	
H8B	0.635720	0.260647	0.421143	0.035*	
H8C	0.654427	0.451599	0.478961	0.035*	
C9	0.15738 (8)	0.6016 (3)	0.29791 (6)	0.0169 (3)	
C10	0.08112 (8)	0.7331 (3)	0.27509 (6)	0.0203 (3)	
H10A	0.084987	0.934742	0.285256	0.031*	
H10B	0.049155	0.638463	0.292905	0.031*	
H10C	0.059499	0.711821	0.232210	0.031*	

### 4-Acetamido-3-chlorophenyl acetate Atomic displacement parameters (Å^2^)

**Table:**

	*U*^11^	*U*^22^	*U*^33^	*U*^12^	*U*^13^	*U*^23^
Cl1	0.02477 (19)	0.0215 (2)	0.01999 (18)	0.00295 (13)	0.00569 (13)	−0.00444 (13)
O1	0.0159 (5)	0.0289 (6)	0.0251 (5)	0.0034 (4)	0.0071 (4)	0.0090 (4)
O2	0.0261 (6)	0.0313 (6)	0.0364 (6)	0.0010 (5)	0.0119 (5)	0.0116 (5)
O3	0.0240 (5)	0.0134 (5)	0.0315 (6)	0.0002 (4)	0.0063 (4)	−0.0009 (4)
N1	0.0171 (6)	0.0118 (6)	0.0186 (6)	0.0003 (4)	0.0039 (4)	−0.0005 (4)
C1	0.0178 (7)	0.0139 (7)	0.0167 (6)	−0.0007 (5)	0.0051 (5)	0.0030 (5)
C2	0.0203 (7)	0.0148 (7)	0.0173 (7)	−0.0006 (5)	0.0069 (5)	0.0011 (5)
C3	0.0186 (7)	0.0208 (7)	0.0167 (7)	−0.0025 (6)	0.0040 (5)	0.0020 (5)
C4	0.0152 (6)	0.0230 (8)	0.0212 (7)	0.0007 (5)	0.0064 (5)	0.0071 (6)
C5	0.0220 (7)	0.0207 (7)	0.0217 (7)	0.0025 (6)	0.0110 (6)	0.0017 (6)
C6	0.0214 (7)	0.0187 (7)	0.0167 (7)	−0.0003 (5)	0.0071 (5)	0.0001 (5)
C7	0.0198 (7)	0.0221 (7)	0.0173 (7)	−0.0017 (6)	0.0073 (5)	−0.0030 (6)
C8	0.0188 (7)	0.0283 (8)	0.0235 (8)	−0.0005 (6)	0.0076 (6)	−0.0014 (6)
C9	0.0189 (7)	0.0158 (7)	0.0155 (6)	−0.0011 (5)	0.0057 (5)	0.0013 (5)
C10	0.0175 (7)	0.0182 (7)	0.0234 (7)	−0.0001 (5)	0.0050 (5)	0.0004 (6)

### 4-Acetamido-3-chlorophenyl acetate Geometric parameters (Å, º)

**Table:**

Cl1—C2	1.7378 (15)	C4—C5	1.385 (2)
O1—C7	1.3692 (18)	C5—C6	1.388 (2)
O1—C4	1.4033 (17)	C5—H5	0.9500
O2—C7	1.1958 (19)	C6—H6	0.9500
O3—C9	1.2271 (18)	C7—C8	1.494 (2)
N1—C9	1.3592 (18)	C8—H8A	0.9800
N1—C1	1.4129 (17)	C8—H8B	0.9800
N1—H1N	0.87 (2)	C8—H8C	0.9800
C1—C6	1.393 (2)	C9—C10	1.5027 (19)
C1—C2	1.3965 (19)	C10—H10A	0.9800
C2—C3	1.389 (2)	C10—H10B	0.9800
C3—C4	1.376 (2)	C10—H10C	0.9800
C3—H3	0.9500		
			
C7—O1—C4	116.09 (11)	C5—C6—H6	119.4
C9—N1—C1	124.31 (12)	C1—C6—H6	119.4
C9—N1—H1N	118.6 (13)	O2—C7—O1	122.79 (14)
C1—N1—H1N	117.1 (13)	O2—C7—C8	126.87 (14)
C6—C1—C2	118.04 (13)	O1—C7—C8	110.33 (12)
C6—C1—N1	121.95 (12)	C7—C8—H8A	109.5
C2—C1—N1	120.01 (13)	C7—C8—H8B	109.5
C3—C2—C1	121.72 (13)	H8A—C8—H8B	109.5
C3—C2—Cl1	118.56 (11)	C7—C8—H8C	109.5
C1—C2—Cl1	119.72 (11)	H8A—C8—H8C	109.5
C4—C3—C2	118.35 (13)	H8B—C8—H8C	109.5
C4—C3—H3	120.8	O3—C9—N1	122.91 (13)
C2—C3—H3	120.8	O3—C9—C10	121.46 (13)
C3—C4—C5	121.84 (13)	N1—C9—C10	115.63 (13)
C3—C4—O1	119.30 (13)	C9—C10—H10A	109.5
C5—C4—O1	118.81 (13)	C9—C10—H10B	109.5
C4—C5—C6	118.91 (14)	H10A—C10—H10B	109.5
C4—C5—H5	120.5	C9—C10—H10C	109.5
C6—C5—H5	120.5	H10A—C10—H10C	109.5
C5—C6—C1	121.12 (13)	H10B—C10—H10C	109.5
			
C9—N1—C1—C6	41.6 (2)	C7—O1—C4—C5	86.39 (17)
C9—N1—C1—C2	−138.86 (14)	C3—C4—C5—C6	0.3 (2)
C6—C1—C2—C3	0.2 (2)	O1—C4—C5—C6	177.91 (13)
N1—C1—C2—C3	−179.30 (13)	C4—C5—C6—C1	−1.2 (2)
C6—C1—C2—Cl1	−179.28 (11)	C2—C1—C6—C5	0.9 (2)
N1—C1—C2—Cl1	1.21 (18)	N1—C1—C6—C5	−179.57 (13)
C1—C2—C3—C4	−1.0 (2)	C4—O1—C7—O2	6.4 (2)
Cl1—C2—C3—C4	178.46 (11)	C4—O1—C7—C8	−172.55 (12)
C2—C3—C4—C5	0.8 (2)	C1—N1—C9—O3	−0.6 (2)
C2—C3—C4—O1	−176.80 (12)	C1—N1—C9—C10	178.72 (12)
C7—O1—C4—C3	−95.97 (16)		

### 4-Acetamido-3-chlorophenyl acetate Hydrogen-bond geometry (Å, º)

**Table:**

*D*—H···*A*	*D*—H	H···*A*	*D*···*A*	*D*—H···*A*
N1—H1*N*···O3^i^	0.87 (2)	2.01 (2)	2.8335 (17)	157.5 (17)
C8—H8*A*···Cl1^ii^	0.98	2.90	3.8279 (16)	159
C8—H8*C*···Cl1^iii^	0.98	2.97	3.6541 (16)	128
C10—H10*A*···O3^i^	0.98	2.46	3.2796 (18)	141

Symmetry codes: (i) *x*, *y*+1, *z*; (ii) *x*+1/2, −*y*+2, *z*; (iii) −*x*+1, −*y*+2, −*z*+1.
